# Initiating therapeutic relaxation in Britain: a twentieth-century strategy for health and wellbeing

**DOI:** 10.1057/palcomms.2016.43

**Published:** 2016-07-19

**Authors:** Ayesha Nathoo

**Affiliations:** 1 University of Exeter, Exeter, UK

## Abstract

In 1972, a British charity, Relaxation for Living, was established “to promote the teaching of physical relaxation, to combat stress, strain, anxiety and the tension of modern life, and to reduce fatigue”. This article explores the origins and development of “physical relaxation” techniques and ideologies, starting in the interwar period, and the development of practical, therapeutic, social and cultural frameworks necessary for such an organization to come into being in 1970s Britain. It traces how relaxation was reconstituted as a scientifically-based skill that could be learnt and taught, imbued with therapeutic value for combating and preventing specific physical ailments and enhancing individual health and wellbeing. The article explores how relaxation techniques gained currency among particular demographic and clinical groups, ranging from middle-class, child-bearing women to middle-aged, “coronary-prone” men. This analysis highlights the role that relaxation practitioners played in both creating and responding to demand for individualistic health-management strategies, many of which have shaped contemporary health and wellbeing agendas. This article is published as part of a collection entitled “On balance: lifestyle, mental health and wellbeing”.

## Introduction

As Amber Lloyd, the founder of Britain’s first relaxation charity—Relaxation for Living (RforL)—reminisced, her husband had suffered a heart attack in 1969 and was told by his doctor to relax. “We said”, recalled Lloyd in an interview for the women’s magazine *Annabel*, “What a super idea. Where does one go to do that? … But of course, it was just a figure of speech. There was nowhere for him to learn to relax”. In response to this seemingly unmet need, Lloyd, who had been teaching relaxation within National Childbirth Trust (NCT) antenatal classes since 1959, set about to make these techniques more widely available. She established Relaxation for Living as a charity in 1972 “to promote the teaching of physical relaxation, to combat stress, strain, anxiety and the tension of modern life, and to reduce fatigue” (Ward, 1980). Underpinning this “origin story” were the assumptions that relaxation was something that needed to be learnt, could be professionally taught, would positively impact on health and wellbeing, and could be exercised in everyday living.

As this article will demonstrate, this ideological framework had been established during the early decades of the twentieth century. It led to several fields, including antenatal care, physical education, speech therapy, psychotherapy, yoga and physiotherapy, all incorporating bodily relaxation techniques for therapeutic purposes. The article will highlight the role of key individuals responsible for developing and applying “neuro-muscular” or “physical” relaxation, and the broader medical and social changes and conditions that made possible the establishment of RforL in 1970s Britain. In doing so, it will bring together areas of medical history that have tended to be explored separately, such as women’s health and childbirth, heart disease and stress research, and preventive medicine. It will also draw out ways in which gender steered the differential uptake of therapeutic relaxation practice. With an emphasis on Britain from the interwar period until the early 1970s, it will complement recent scholarship on “stress”, mainly focussed on the United States (for example, Harrington, 2008; Jackson, 2013), and provide an historical understanding of contemporary health and wellbeing frameworks.

Implicit and recurrent in this history of relaxation therapy is the concept of “balance”. Key therapeutic goals of relaxation have been to balance overactive minds and bodies, including hormonal and immunological change and physiological stress responses deemed to be detrimental to health and wellbeing. As Lloyd (1981: 1–2) explained, a relaxed person is essentially a balanced person:

Every human being is made up of three parts—mind, emotion and body. When we speak of ‘a relaxed person’, we mean someone who functions efficiently, with all three parts working in harmony. Between these three human parts there is very close interaction. If any one of them is upset, the other two are thrown out of gear, the balance becomes disturbed and working ceases to be smooth and harmonious.

In sum, “Life (and staying alive)”, Lloyd remarked, “is always a matter of compromise and balance. These are not only philosophical concepts, they are physiological facts” (Lloyd, 1977: 1).

This article will explore how relaxation became a principle strategy for achieving and maintaining “balanced”, healthy lives. It is divided into four sections. The first part outlines the development of “physical” relaxation practices in the interwar period, starting with the work of the Chicago physician Edmund Jacobson (1888–1983). It explains how relaxation ideology was appropriated in Britain, and applied therapeutically to help with specific ailments, nervous illness and for reducing tension in everyday living. The second section analyses the central area in which relaxation practices developed in Britain—for pre- and postnatal, mainly middle-class, women. It highlights the place of obstetrician Grantly Dick-Read (1890–1959) in relaxation history and the relaxation training that constituted and then grew out of antenatal class teaching. The third part explicates how relaxation was drawn into prevailing postwar concerns regarding heart-disease prevention and rehabilitation, particularly for middle-aged working men. The last section details the establishment of RforL as a charity in 1972 and the consolidation of relaxation as a therapy, prophylaxis, art, science, skill and way of life, intimately connected to good health and wellbeing.

## Redefining relaxation in the interwar years

The methods of therapeutic relaxation taught in the 1970s by RforL were in the most part devised in the interwar years in both Britain and the United States—a period defined by evident and feared hardship and decline (Overy, 2009). With populations still recovering from the consequences of the First World War, the 1930s global depression had a devastating effect on employment, birth rates were falling and the “fitness” of nations was being called into question (Zweiniger-Bargielowska, 2010). Although the medical diagnosis of “neurasthenia” (a term popularized by New York neurologist George Beard (1839–1916) in the 1880s) had fallen out of favour in the interwar period, complaints of nervous illness and exhaustion persisted unabated (Gijswijt-Hofstra and Porter, 2001). In this broad context, relaxation was presented as an antidote. It was a skill that could be learnt, cultivated and applied for therapeutic effect, to prevent and alleviate fatigue and tension linked to the strains and frustrations of modern living.

The Chicago physician and psychologist Edmund Jacobson was highly influential in establishing this therapeutic framework and practice. Working on the premise that one cannot be tense and relaxed at the same time, Jacobson devised an elaborate method of muscular relaxation that involved systematically recognizing and releasing all unnecessary bodily tension. “It is physically impossible to be nervous [or tense] in any part of your body, if in that part you are completely relaxed”, he declared (Jacobson, 1934: 43). Jacobson proposed that both thought and emotion produced muscular tension, so the subjective experience of an emotional state such as anxiety was expressed within strongly contracted muscles. Therefore, he asserted, physically relaxing would reduce emotional as well as bodily tension.

Patients learnt his techniques of progressive and differential muscle relaxation through an intense teaching programme involving two or more hours instruction per week plus practise alone of one or two hours each day (Jacobson, 1934: 126). Relaxation, he proposed, was superior to “rest” or even sleep, given that the body could remain tense during resting periods: this being the reason for the “many failures which are commonly reported concerning the so-called ‘rest-cure’ of Doctor Weir Mitchell” (Jacobson, 1934: 44). Representative of a wider disciplinary movement to transform psychology into an empirically based experimental science, Jacobson developed an electromyograph, or “neuro-voltmeter”, capable of detecting tiny changes in electrical activity within muscles (Kroker, 2003). This rendered tension detectable, measurable, visible and, most importantly, demonstrated that it was diminishable through skilled practice of his neuro-muscular relaxation technique.

Jacobson first presented his ideas in a technical book aimed at his fellow medical and scientific practitioners *Progressive Relaxation: A Physiological and Clinical Investigation of Muscular States and their Significance in Psychology and Medical Practice* (Jacobson, 1929), and then in his 1934 popular book, *You Must Relax: A Practical Method of Reducing the Strains of Modern Living*. He set about redefining relaxation, away from its vernacular usage associated with “amusement, recreation or hobbies”, instead to imply “neuro-muscular”, “cultivated” or “scientific” relaxation that required skilful teaching and practice. Jacobson suggested that “neuro-muscular hypertension” could substitute the term “neurasthenia”, and was in fact responsible for “so-called neurasthenic symptoms” (Lanier, 1930: 473). Relaxation, he proposed, was “a direct and specific treatment” for the “overfatigued” or what was “frequently called ‘nervousness’ ”. He advocated relaxation’s wide therapeutic implications for a range of clinical conditions and more generally for “the strains of modern living” (Jacobson, 1934). Relaxation, he determined, would benefit people across occupations, including businessmen, lawyers, doctors, teachers, factory-workers, salesmen or women, telephone operators and “even the farmer, striving to pay taxes and interest”. Acknowledging that the “rearing of children still takes its toll from the nerves of the watchful mother”, and that “youngsters” too, from infancy through adolescence, suffer from the “overstimulation of modern times”, he proposed muscle relaxation as an effective scientific method applicable and useful for all to enjoy “modern civilization without burning the candle at both ends” (Jacobson, 1934: 8).

Jacobson made an effort to place his relaxation techniques firmly within the Western, secular, scientific landscape and clearly distanced his relaxation principles from the hypnotic tradition and forms of mysticism. He demarcated his teachings from that of the German psychiatrist Johannes Schultz (1884–1970) who, at a similar time, was developing “Autogenic Training”—a relaxation technique based on visualization methods with clear yogic influences (Lehrer, 1996; Singleton, 2005). Relaxation training had already gained popularity within gymnastics and the performing arts at the turn of the century, particularly under the guidance of New Thought author, Annie Payson Call (1853–1940) in Massachusetts (Capps, 2009; Andrick, 2012). While commending the practical elements of Call’s teachings, helping people to cultivate poise and use relaxing exercises, Jacobson lambasted Call as a “cult” follower of Swedenborg, claiming that “her interests are not scientific and when she states that an individual may remain nervous while relaxed by her measures, it is evident that she fails to study the extreme or finely drawn out relaxation” that was the “essential aim” of his own method (Jacobson, 1934: 44–45).

In interwar Britain, the teachings of both Jacobson and Call became influential. Frances Archer (1855–1926), wife of playwright William Archer (1856–1924), was largely responsible for introducing Call’s methods in the British context after learning relaxation exercises directly from her in Massachusetts. Before the First World War, Archer established a “Nerve Training Colony” in King’s Langley, Hertfordshire, where people could go to “recuperate from the hurly-burly of everyday life” (Caton and Archer, 1936). Many residents here were thespians and relaxation training was used to calm the nerves and help with specific speech disorders such as stammering. The performing arts were one of the first arenas to adopt relaxation training; Call’s own background had been in acting and her teachings were inspired by Delsartism, which had enjoyed widespread popularity among late nineteenth-century middle-class American women (Andrick, 2012). In this tradition, relaxation was not linked to stillness, but, as one of FM Alexander’s (1869–1955) first students later declared, was inseparable from “well coordinated movement, grace and poise” (Neil, 1958).

In the 1930s, relaxation teachings were disseminated to a wider population, notably within antenatal instruction, speech therapy and through popular books. London-based relaxation classes for stammering children were led by EJ Boome, Principal Assistant Medical Officer at London County Council, and MA Richardson, a lecturer and speech therapist at the Elizabeth Garrett Anderson Hospital. In their 1938 publication, *Relaxation in Everyday Life*, “intended primarily for the man in the street”, Boome and Richardson, warned readers that although they sought to avoid as far as possible “technical terms and polysyllabic elaborations”, therapeutic relaxation was “*not* a simple technique to learn”. It required much practice given that “modern civilization and the high speed of present-day living are leading us further and further from fundamental needs and realities” (Boome and Richardson, 1938: 1). They noted the trouble in “making clear what is understood by the term ‘relaxation’ ”, given that one person would think it suggests “a holiday”, another “a thoroughly lazy time in a deck-chair, a punt, or a hammock”, and for someone else it would be playing “a round of golf”. Relaxation as they construed it was “much more far-reaching in its effects and more constructive in its processes, and, beginning with a purely muscular loosening, leads to a gradual re-creating of the individual”. It was a “self-treatment”, an “art” which needed to be “deliberately learnt” and practised in everyday life to counteract tension expressed in “muscular hypertonicity, in mental worry, anxiety and fear, in emotional repression and nervous strain”. “The balance and stability which can be obtained by the practice of relaxation”, they told readers, “enables a man to face and deal fearlessly with responsibility and trouble” (Boome and Richardson, 1938: 3; 99).

Boome and Richardson deemed relaxation beneficial for a number of “disorders”—including insomnia, enuresis, masturbation, dysmenorrhoea, menopause, asthma and stammering—but were chiefly concerned with relaxation that was both “practicable” and “desirable in everyday life”. They differentiated between deep, restorative relaxation, best conducted lying down and requiring suspension of usual activity, and relaxation that could be done while being active. The latter was not the “complete process”, but it would serve to conserve energy and reduce unnecessary tension: “Could you not write that letter just as well without clenching the other hand, and your jaw? Without bracing your thighs and gripping the floor with your feet?”, they asked. “Alter your position and sit properly ON your chair, instead of hovering above it … Make a habit of letting your legs ‘go off duty’ whenever you sit down” (Boome and Richardson, 1938: 19). This tallied with Jacobson’s system of “differential muscle relaxation”, for use in everyday activities, whereby only muscles essential to the task at hand should be tense and the rest of the body was to relax. And as with Jacobson, Boome and Richardson advocated a form of relaxation, which once learnt correctly could be applied by anyone, anywhere.

Relaxation practice proliferated in Britain during the postwar decades, and by WW2 it was already starting to be applied within clinical and non-clinical settings, with wide implications for health and wellbeing. Ranging from speech therapy to calming the nerves, from counteracting exhaustion to managing pain, relaxation was promoted as a safe and effective skill that could be learnt and cultivated for therapeutic effect for specific conditions and for enhancing personal and communal everyday living. Jacobson was undoubtedly foundational and influential, but the relaxation ideology that was promulgated in Britain equally borrowed from the teachings of Annie Payson Call, via Frances Archer. As the next section will demonstrate, the work of the obstetrician Grantly Dick-Read and the NCT, which grew out of his teachings, were also central to the proliferation of relaxation therapy, especially among middle-class women. In mid-century Britain, childbirth became the primary area where relaxation training was systematized and therapeutically applied.

## Helping women to help themselves: relaxation for childbirth, relaxation for life

In the postwar years, relaxation came to have an important association for middle-class women, which focused on their ability to successfully fulfil their social roles and responsibilities as mothers. Relaxation training, to a large extent, both established and transformed the field of antenatal care. The development of group classes delivered by professional, but non-medical, teachers of relaxation, was the model later used by RforL when it started in the 1970s. Influenced by the eugenically-inspired pronatalism of the time, the British obstetrician Grantly Dick-Read theorized that middle-class women were not reproducing enough because of their culturally induced fear of the pain of childbirth (Moscucci, 2003). Caught in this fear-tension-pain cycle, Western women had lost their “natural” ability to give birth free of these debilitations. Relaxation, Dick-Read proposed, was a way in which to break the cycle, and allow for a “natural childbirth”—a term he coined in his 1933 exposition of labour pain (Dick-Read, 1933). Dick-Read was aware of Jacobson’s techniques, and credited an Indian serviceman for introducing him to the merits and practice of relaxation to manage fear during the war (Noyes Thomas, 1957: 60).

Whereas Dick-Read’s own relaxation teachings were largely philosophical and theoretical, contemporary midwives and physiotherapists, such as Minnie Randell, Helen Heardman and Kathleen Vaughan, devised practical methodologies of relaxation, mainly aimed at middle-class child-bearing women (Moscucci, 2003; Hay-Smith, 2013). Randell, a nurse, midwife and the principal of the school of physiotherapy at St Thomas’ Hospital, from its establishment in 1911 until 1945, laid the foundations of the field of obstetric physiotherapy in Britain. She developed antenatal and postnatal classes incorporating relaxation exercises, inspired by the teachings of Archer and Jacobson, and made relaxation instructions accessible outside of clinical settings through popular books designed to help women to “help themselves” (Randell, 1939, 1948).

After the establishment of the NHS in 1948, and the wartime manufacture of penicillin—the archetypal “magic bullet”—science and medicine prospered in Britain’s postwar “white heat of technology”. Despite loud calls for implementing a more holistic model of “psychosocial medicine” (Hayward, 2009, 2012), state hospitals were largely driven by technological and pharmaceutical optimism and intervention that privileged treating individual illness and disease. One area that underwent significant change was childbirth. In the postwar period, hospitals became key sites for childbirth and women found medical technologies and personnel increasingly dominating their birth experiences. Although many women celebrated, indeed campaigned for, increased access to analgesics and the availability of hospital care, others found the experience disempowering and even threatening (Marland, 2000; Al-Gailani, forthcoming). For many of these women, Dick-Read’s philosophy presented a desirable, viable alternative.

On 4 May 1956, following the traumatic stillbirth of her son at a London hospital, and seeking an alternative framework for childbirth, a young woman named Prunella Briance placed an advertisement in *The Times* calling on like-minded mothers to help establish an organization to promote the teachings of Dick-Read. This marked the beginnings of the National Childbirth Trust (NCT), originally set up in 1956 under the name of the Natural Childbirth Association (Kitzinger, 1990). Sidelined by his professional colleagues and never granted membership of the Royal College of Obstetricians and Gynaecologists, Dick-Read’s work might have diminished in relevance, were it not for the establishment of the NCT gaining him widespread recognition and support from a network of laywomen (Thomas, 1997). The teaching of relaxation exercises in the antenatal period was a central component of NCT membership and formed an integral part of efforts to positively transform the experience of parturition, in line with Dick-Read’s philosophy.

Amber Lloyd, who later established RforL, was one of the founding members of NCT, and had one of her children delivered by Dick-Read himself. Briance encouraged her to establish the first local NCT branch, at Walton and Weybridge, which she successfully managed for over 15 years. In the early years of NCT, when Dick-Read was Chairman, he insisted that all technical instruction be based on the teachings of his second wife Jessica, who had devised a programme of antenatal instruction. Lloyd herself had produced a popular leaflet on breathing instruction which Dick-Read bitterly opposed being distributed as it was not under his authorship. Notes to NCT teachers outlined numerous rules including that men were not allowed in the classes as this may be distracting for the proper practice of breathing and relaxation techniques (“Notice to NCT Area Organizers” (1957) reproduced in Kitzinger, 1990: 103).

Dick-Read died in 1959, and a tumultuous period followed for the organization. When Briance was temporarily living in the United States, NCT formally changed its allegiance towards the psychoprophylactic method of Fernand Lamaze (1891–1957), no longer privileging Dick-Read’s teachings. Briance, being fiercely loyal to Dick-Read whose ideology laid the foundation of NCT, was later pushed out of the organization.^1^ Although Lamaze’s theoretical framework based on conditioning was quite different to Dick-Read’s, antenatal relaxation instruction was central to both approaches (Michaels, 2014). Moreover, the framework of antenatal teacher training remained intact in allowing and encouraging laywomen to train as NCT antenatal instructors. Some of those instructors had experience of working, for example, in midwifery, nursing or physical education, but many, such as Amber Lloyd, had no previous clinical or teaching background. Although NCT membership was available to all women, by and large it was comprised of middle-class women, especially those who could afford to volunteer their time at the teaching and organizational level.

In the years following Dick-Read’s death, despite the reorientation towards Lamaze’s teachings, NCT instructors taught a far broader range of techniques and learnt from a wide range of approaches, many of which were imparted during regular “study days” for teachers. Lloyd succeeded in developing one of the strongest local branches of NCT, which enjoyed a fair amount of autonomy. She produced a branch newsletter, and established close connections with local medical professionals, in line with NCT’s general policy of working alongside, rather than against the medical establishment in this period (Kitzinger, 1990: 105). Her experience at NCT was later to provide the structural and teaching model for RforL, which she set up together with fellow NCT colleagues and “advanced” antenatal teachers. Like NCT, RforL developed study days for ongoing teacher training, produced regular newsletters, and strengthened ties with local GPs and other medical professionals to lend support to group relaxation classes taught by non-medical teachers.

By the 1960s, a number of antenatal teachers, including Lloyd herself, were starting to extend relaxation teachings from the narrow context of childbirth to help women manage their wider experiences of motherhood. As another NCT founder member, Betty Parsons, summed up in her motto: “Relaxation for childbirth, relaxation for life!” (Parsons, 1996). In Birmingham, Jane Madders, a physical education teacher, physiotherapist and antenatal instructor (later to become a key figure in the establishment of RforL) had started a “Family Club” in 1952, claimed to be the first “playgroup” in the country, where children could play and mothers could continue to learn relaxation to help with their lives after their babies were born (Madders, 1955; Bates, 1979).

Relaxation teachings also became available to women through yoga as it spread to the West in the postwar decades. As yoga historian Singleton (2005) has argued, “Modern Yoga” was itself reconstituted in the West by incorporating “relaxationism”, owing more to the teachings of Call and Jacobson, for example, than to any ancient Eastern tradition. Yoga, like Transcendental Meditation (TM), swept to popularity in the “swinging sixties”, but was by no means restricted to counter-culture followers with an interest in Eastern spirituality; instead, as Newcombe (2007, 2014) has demonstrated, it was practised primarily by middle-class housewives in Britain—the same demographic as the NCT membership. With a focus on poise, posture and breathing, relaxation also continued to be practised as preparation for the performing arts, including within the intricate system developed by FM Alexander and his students (Neil, 1958). New methods of relaxation were also developed and applied within the fields of physiotherapy, occupational therapy, physical education and health education, and increasingly in psychotherapeutic settings (for example, Garmany, 1952; Wallace, 1965; Mitchell, 1977).

In addition, relaxation advice and instruction could readily be found within the growing self-help literature, and accessed through radio programmes and the new medium of domestic television. Many of these programmes primarily targeted women, especially housewives, such as Radio 4’s *You and Yours* and *Woman*’*s Hour* and BBC1’s daytime show *Pebble Mill at One*. In these programmes, relaxation was presented as a tool to help with an array of nervous disorders afflicting the modern-day woman, and an alternative to the widespread use of tranquillisers such as Valium.^2^

Therefore, by the time RforL was established in the early 1970s, the demand for therapeutic relaxation, especially among middle-class women, had been firmly established, starting with antenatal care. As a set of techniques and ideologies, therapeutic relaxation then extended into all aspects of managing and bettering modern experiences of motherhood and womanhood more generally. As the next section elucidates, relaxation entered the lives of men through different channels, primarily through developing an association with heart disease prevention and as an antidote to “stress”.

## Appealing to men: relaxation for a healthy heart

One of RforL’s most widely distributed teaching materials was a series of pamphlets authored by Lloyd, including *Why Rush to Raise Your Blood Pressure, Ruin Your Heart, or Have a Stroke?* (Lloyd, 1977). This pamphlet specifically called for the “most valuable members” of modern society whose “intellectual capital is too precious to squander”, and “business executives” who are “so busy ensuring that they waste no time that they waste their whole lives”, to take control of their health by learning and practising relaxation. Readers were told:

The truth is shocking … The choice is yours … A large proportion of the cases of high blood pressure, heart attacks and strokes are partly self-inflicted and avoidable.

The pamphlet warned of the dangers of “being driven at full throttle without any respite”. Such concerns about the pace of modern life and its effects on different social strata were by no means new (Schaffner, 2014), but the focus on deriving causal links between lifestyle and heart disease, and finding new methods to reduce it was a key postwar concern. In this period, cardiovascular disease overtook tuberculosis as a greater threat to Western lives and was considered to be a problem particularly for the modern male worker.

By the 1950s, in Britain, heart disease had become the nation’s “biggest killer”. In 1969 the World Health Organization (WHO) warned that heart disease could soon present mankind with the greatest epidemic it has ever faced (World Health Organization (WHO) Executive Board, 1969). Attempts to manage and curtail it ranged from high-tech, pioneering open-heart surgery, including the sensational heart transplants of the late 1960s (Nathoo, 2009), to lower-key public health campaigns aimed at altering lifestyles and focusing on preventive measures. The Framingham heart study, and the Seven Countries study in the States, as well as MRC studies in Britain, developed through the nascent field of chronic-disease epidemiology, helped to forge the links between the prevalence of heart disease and exercise, diet, smoking, high blood pressure and other “risk factors” (Timmermann, 2011; Aronowitz, 2012). Early proponents of social medicine pointed to such large-scale population studies as indicative of the links between health, social conditions, poverty and inequality. But by the 1960s, the field of social medicine was reorienting from relating ill-health to social structure towards a focus on patterns of behaviour (Porter, 2001).

Pragmatic, risk-based, individualistic preventive and therapeutic solutions became favoured in Britain’s “new public health”, especially following the 1962 report by the Royal College of Physicians, which linked smoking to lung cancer (Berridge, 2007). Reflective of wider social shifts, the risk factor approach led to a reconceptualization of heart disease as being in the most part preventable if responsible individuals chose to alter their “lifestyles” and consumption habits. Heart disease was no longer an inevitable process of aging and degeneration, as it had largely been conceptualized at the turn of the twentieth century, but was instead a disease both caused and obviated by an individual’s ways of living (Weisz, 2014). As epidemiological data made visible, the “middle-aged” were a key “vulnerable group in Western Society” (Morris *et al.*, 1963: 70). Consuming less fat, exercising more and smoking less, were some of the ways in which individuals, especially within this demographic, were encouraged to help themselves. In this context, relaxation was promoted as an effective addition to the self-care toolbox. It was deemed especially relevant to men’s health through the construction of hypertension as a key “risk factor”, the framing of middle-aged working men comprising the most “at risk” population, and the developing discourse of “stress”.

Until the 1960s, “hypertension” had been a disputed medical category, and the term was not used exclusively to relate to blood pressure. Writing to a general audience in the 1930s, Boome and Richardson used the term to denote a state of being extremely tense (hyper-tense) or very anxious, and Jacobson too had referred to “neuro-muscular hypertension”. With reference to high blood pressure, in the nineteenth century this was a condition believed to predominate among women. By the early twentieth century, alongside other psychosomatic conditions that underwent a “sex shift”, it came to be seen as a male disease but was by no means a stable diagnostic category (Hayward, 2012: 289). As Timmermann (2006b) has outlined, starting in the late 1940s a decade-long controversy played out between pioneering British cardiologists Robert Platt (1900–1978) and George Pickering (1904–1980) in the pages of the *Lancet*. The debate, subsequently referred to as the “battle of the knights”, centred on two different understandings of hypertension as either a discrete illness category, “essential hypertension”, or a trait distributed on a continuum (Littler, 2000: 312). On the latter view, which ultimately prevailed, a new population of “at risk” individuals were created: potential heart disease patients, with “mild hypertension”, whose illnesses could be averted through prescription of the newly developed, heavily marketed, hypertensive drugs (Timmermann, 2006a). With this model in place, by the 1970s, when RforL was established, relaxation could be advocated as a life-long drug alternative, without negative side effects, and moreover with a wealth of other benefits to health and wellbeing.

In the early twentieth century, founders of the “new cardiology” in Britain, such as James Mackenzie (1853–1925) and Thomas Lewis (1881–1945), had recognized the impact of harmful psychological and environmental influences on the functioning of the heart and the wellbeing of the individual (Lawrence, 1985). Comparable to causes of “shell-shock”, wartime strain and exhaustion was also seen as responsible for the condition of “soldier’s heart” or “effort syndrome” as it was labelled in Britain. This was reframed as a psychological rather than cardiac problem, which could be treated with graded exercise or rest (Howell, 1985). But early twentieth-century physicians, including William Osler (1849–1919), also recognized that the pace and strains of everyday life, even during peacetime, could pathologically affect the hearts of their patients—and certain groups were more susceptible than others. In *You Must Relax*, Jacobson (1934: 33) built on Osler’s claims from 1910 that “the ordinary high-pressure business or professional man suffering from angina pectoris may find relief, or even cure, in the simple process of slowing the engines”. If the problem arose from the individual’s pace of life, the solution also lay with the individual—to learn to slow down, through cultivating the skill of relaxation.

Postwar psychological frameworks furthered the notion that certain “types” of individuals or “personalities” were more or less vulnerable to ill-health and heart disease. In 1974, cardiologists Friedman and Rosenman’s notion of “Type A” personalities being most coronary-prone was widely publicized through their popular book *Type A Behavior and your Heart* (Friedman and Rosenman, 1974). But these ideas had been in circulation, and highly debated among clinicians since their 1959 scientific publication on this topic, delineating Type A behaviours to include extreme ambition and impatience, and a range of other typically “masculine” character traits (Riska, 2000). Their work contributed to the promotion of psychosocial models of health and disease with associated therapeutic interventions, and the stereotyping of coronary-prone, middle-aged male business executives. In the early 1960s, the Chest and Heart Association of Great Britain ran a series of conferences specifically on the “health of executives”, who were singled out as the group most in danger of cardiac disease as a result of the “stress” of everyday living. Although the landmark Whitehall studies of British civil servants demonstrated that the social gradient of cardiac disease had in fact reversed by the 1950s—that is, workers of lower social standing were more at risk of developing heart disease than their middle-class counterparts (Marmot *et al.*, 1978)—the stereotype of the “at-risk” business executive endured well into the later decades of the twentieth century.

“Stress” research, in particular the Hungarian-born, Canadian physiologist Hans Selye’s (1907–1982) biological formulation of stress, and the American psychologist Richard Lazarus’ (1922–2002) articulation of “coping” mechanisms, gained traction in the postwar decades and further boosted this rationale. In turn it enabled relaxation training to be marketed as an empowering addition to an individual’s armoury that could strengthen one’s ability to cope in stressful environments. Stressful conditions in themselves were not the problem, rather an individual’s relationship with and to those conditions. Jacobson’s, 1963 publication: *Tension Control for Businessmen*, and other popular books such as *Relaxation for Men: Tension in Modern Living* (Brierley, 1965) and *Do Something about those Arteries* (Sears, 1968), advised on reducing worry, tension and stress in everyday living through relaxation, especially for the sake of men and their hearts. Belief that the strain of modern living took its toll on physical and mental health was not particular to the middle decades of the twentieth century, but the growing discourse of “stress” allowed for a rapid proliferation of this idea and the development of associated therapeutic interventions, such as relaxation.

As recent historical scholarship has demonstrated, the notion of “stress” that was developed and relentlessly promoted by Selye, built on a wide range of prior scientific research. This included the American surgeon George Crile’s mechanistic and adaptive theories of disease, and physiologist Walter Cannon’s pioneering work on “homoeostasis” and his delineation of the “fight or flight” response (Jackson, 2013). Nonetheless, Selye’s notion of the “General Adaptation Syndrome” and his 1956 publication *The Stress of Life*, contributed considerable scientific credence to pre-existing notions of psychopathology linked to the chronic strain of everyday modern living. Allied to the growing field of psychosomatic medicine, stress became seen as both the cause and the consequence of a host of physical illnesses, including heart disease and hypertension (Jackson, 2007: 161). As “stress” became more scientifically and physiologically valid, and ever more diverse and flexible in its meaning, helping to “blur the distinction between physical and psychological illness” (Cantor and Ramsden, 2014: 7), so too did relaxation—as an antidote. As Watkins (2014: 64) has noted with regards to popular magazine articles on stress, by the late 1970s, “Whatever the cause of the stress and whoever suffered from its effects, the advice given—usually at the end of the article or in an accompanying column—was consistently similar: relax”.

As well as a preventative measure for stress and heart disease, by the 1970s relaxation strategies also became applicable to the rehabilitation of those who had already developed heart disease—in terms of averting a future “coronary” and adjusting to the psychological consequences of cardiac disease. As previously discussed, central to Lloyd’s interest in establishing RforL was her husband’s heart attack, and subsequent advice from his doctor to relax. As she recalled in an interview in 1980, while her husband had not found anywhere to learn to relax, he had been to “courses at the City Gymnasium in London” where he had undertaken a supervised exercise plan (Ward, 1980). Exercise initiatives such as these were only just starting to be trialled in the United Kingdom within the nascent field of cardiac rehabilitation. Since the Second World War, rehabilitation in Britain had primarily been focussed on physical handicaps and getting people back to work, and compared with continental Europe and the United States, cardiac rehabilitation advice and services were slow to start (Weisz, 2014: 200). Until the 1940s, prolonged bed rest comprised the traditional treatment for those who survived a coronary thrombosis, but this started to be questioned by some cardiologists who instead promoted mobilization. Physical complications, such as “infarction of the lungs, pneumonia and uremia” that often followed bed rest, were reduced through early mobilization, which also reportedly provided psychological benefits of reducing “cardiac neurosis”. The psychological impact of cardiac problems on quality of life were, however, less acknowledged within rehabilitation practices developed during the 1950s and 1960s, which concentrated primarily on clinical treatment and the physical aspects of mobilization (Stokes, 2000). In 1969, the WHO provided a multifaceted definition of cardiac rehabilitation referring to “physical, mental and social conditions”, as well as the importance of individuals taking responsibility for their own health needs (World Health Organization (WHO) Regional Office for Europe, 1969).

This broader perspective allowed for relaxation, alongside exercise to be seen as an important contributor to improving physical, mental and social wellbeing. Contrary to Lloyd’s assertions that there was nowhere for her husband to learn to relax, by the early 1970s there were a number of arenas where relaxation instruction was already found, ranging from physiotherapy to the burgeoning self-help literature. Nonetheless, conditions were ripe for establishing an organization solely devoted to teaching men and women how to relax, and reaffirming and contributing to ideological frameworks developed since the interwar period for why they should do so for the sake of their minds and bodies, work and personal lives.

## Enroling pupils of relaxation—the establishment of Relaxation for Living

The publication of *Future Shock* by the left-wing writer Alvin Toffler, an acquaintance of Selye, ushered in the 1970s (Toffler, 1970). Drawing on decades of stress research, Toffler postulated that individuals were unable to adjust fast enough, socially and psychologically, to the rapid technological change, “information overload”, and “sick social structure” (Jackson, 2013: 224–27). The bestselling book, and documentary film, released two years after the book, underscored the prevalent malaise in the West derived from a deeply unstable and uncontrollable world caught in the Cold War dread of nuclear annihilation. Although directed at Americans in particular, Toffler’s postulation of “the disease of change” was equally recognizable amongst British populations. Toffler’s concerns echoed some of those expressed by the social anthropologist Edmund Leach, Provost of King’s College Cambridge, who delivered the 1967 BBC’s Reith Lectures, entitled *A Runaway World?* His third lecture, “Ourselves and Others”, opened with: “*Z Cars* and *The Avengers*, film posters, stories of sudden death, fables of Hiroshima: we are surrounded by themes of violence from the day we are born. It is not just nature and technology that seem out of control, it is ourselves” (Leach, 1968: 31). Even before the 1973 Yom Kippur War and subsequent oil crisis that sparked a global economic downturn, this period was characterized by individual, social, political and environmental insecurity. For Toffler, healing the inhabitants of sick societies required a multifaceted approach, involving individual and sociopolitical action: one contributory approach was “the provision of personal strategies for stress reduction and relaxation” (Jackson, 2013: 228).

Appropriately timed, in July 1971 under the name of Relaxation for Living, Lloyd began to teach relaxation classes from her home in Walton-on-Thames. She started with five students and offered a 6-week course, for 1.5–2 hours, one evening per week. The course fee also included use of a small lending library. By November 1971, demand had risen to warrant her offering an extra class a week, this time in the morning. During the first year, 75 people had enrolled in her classes, including people from New Zealand, Canada, America, France and Australia. These early relaxation students comprised the primary readership for the organization’s first newsletter, circulated for free a year after starting, in July 1972. In this, Lloyd disclosed that of the first-year cohort, “several were in the aircraft industry, more were school teachers; one was a Health Visitor, one a midwife and several exnurses”, to which she noted: “I feel this sheds an interesting light on the most stressful occupations of today!”. The largest group, however, were “mothers looking after growing families”; the majority of participants were aged between 30 and 60, although the full range was 16–79. Although women had outnumbered men, the percentage of men in the classes was “creeping up slowly”. As to the motivations for attending classes, “they came for a very wide variety of reasons, all based fundamentally on tension”. Lloyd had also started a “make-shift correspondence course” for “particularly needy enquirers”, sending them weekly class notes, and exchanging written feedback. Her fee for the course was initially £3 but she was open to “pensioners and genuinely hard-up cases” negotiating a lower fee with her “by special arrangement” (Lloyd, 1972).

At the time of writing the first newsletter, Lloyd was trying to register RforL as a charity, with the hope of fundraising, buying more equipment and launching more classes. It was a challenging process, and although the Charity Commission had accepted the organization, Inland Revenue required more evidence. In a postscript labelled “urgent” at the bottom of the newsletter, Lloyd asked for help from past students with addressing some further queries regarding RforL achieving charity status. She explained that Inland Revenue were asking for “evidence that we are running a properly organised progressive course of classes, and not just having coffee and a chat”, and “whether we are organising Relaxation for Living purely to make a comfortable income for the teachers”. Readers were asked to put it in writing if they felt they had received value for their money for their classes, and any other comments that would help to submit as evidence. More of a challenge, given the ideological underpinning of the organization was that: “In the eyes of the law, it is not charitable to prevent illness, breakdown, depression, migraine, what-have-you, but it is charitable to try to cure it after it has happened, and to stop it recurring. Inland Revenue want evidence that we are doing this”. To her newsletter readers, she requested: “If any one of you feels that you were helped to recover from any physical, mental or emotional complaint, please, would you write me a note, giving details, that I could send them?” (Lloyd, 1972).

Many past students did submit supportive testimonies, and soon after circulating the newsletter, towards the end of 1972, RforL succeeded in acquiring charity status. The founding committee included Lloyd as Honorary Secretary and five teaching officers who were fellow NCT “advanced teachers” (who ran classes in Camberley, Guildford, Oxford, Richmond and Chorleywood). She also had the support of four trustees: Katharina Dalton, a gynaecologist pioneering research into hormonal imbalance, premenstrual syndrome and the menopause; Madge Moore, a local doctor who specialised in marriage guidance and family welfare; Peter Wilson, who was Honorary Secretary of the British Migraine Association; and John Pringle, a retired bank manager and one of her first private students, who agreed to become Honorary Treasurer.

Physiotherapist and health educator Jane Madders was the key inspiration behind the organization’s therapeutic ambitions and practical set up. Madders acted as a technical advisor, leading the training of RforL teachers and later became Chairwoman of the organization. Madders had learnt relaxation techniques during her training in the late 1920s at Chelsea College of Physical Education, although she later recalled that at that time, in 1927, the techniques “were only applied in the physical sense. We never learnt how they could affect our mental states as well” (Bates, 1979: 10). She had also been a student of FM Alexander and had known Dick-Read, who, together with midwives Minnie Randell and Helen Heardman, greatly influenced Madders’ own relaxation teachings (Madders, 1955). Early in her career as a physiotherapist, after meeting Dick-Read, she established relaxation antenatal classes at Lordswood Maternity Hospital in Birmingham. Through the “Family Club” that she established in the early 1950s, she extended relaxation teaching to postnatal mothers, who had told her that they needed the techniques even more after their babies had been born. The women who met through the Family Club in the 1950s continued to meet to exchange advice and experiences as grandmothers in the 1970s. Madders had also taught relaxation to trainee teachers, athletes, as well as children in schools and in child guidance clinics. Her more recent work, since the early 1960s, which was beginning to receive widespread recognition, was on the effect of relaxation therapy on migraine sufferers (Hay and Madders, 1971).

In January and February 1972, Madders led a “Training Course in Relaxation Theory and Practice” at the NCT headquarters in London, “for those experienced in relaxation techniques”. It was advertised as a “training course for those wishing to use their knowledge of relaxation for conditions where stress is a contributory factor”. The course, spread over two full days and three evenings, provided practical training sessions and discussion and demonstration of different methods of relaxation; an overview of recent research into relaxation therapy and “recent advances in our understanding of the effect of stress on the physical and biochemical reactions of the body”; the organization of class work and group discussion; counselling, and “relations with the medical profession and other professional bodies”. The course also included a showing of a film and a list of “Useful background reading”, which listed publications by Jacobson, Selye and Lazarus, among other technical articles on behaviour therapy, migraine and pain.

For Lloyd, this study day greatly influenced how RforL classes were structured and taught. As she reassuringly wrote in her first newsletter to RforL students, attendance at the course meant that “We came away feeling we really knew what we were doing now!” (Lloyd, 1972). As well as providing sound pedagogical guidance and information on diverse therapeutic applications of relaxation, Madders’ connection to RforL was also crucial for publicizing the organization’s aims and services nationally. Coinciding with the launch of RforL as a charity in 1972, Madders broadcast a series of six talks, *Relax*–*and Enjoy it*, for introducing relaxation into everyday life, on Radio 4’s daytime *You and Yours* programme. It was hugely popular and was repeated on radio the following year alongside a book, *Relax: The Relief of Tension Through Muscle Control*, written by Madders and published by the BBC together with an associated cassette recording (Madders, 1973).

The foreword to the book, written by the series’ producer, opened with a quote from “one of many letters received” when the relaxation series was broadcast: “For as long as I can remember I’ve been told ‘Relax’, but no one has ever told me how!”. In a similar fashion, Madders opened the introduction to her book with: “ ‘Relax!’ It is likely that someone has said this to you or you have urged someone else to do it and with little effect. Although we are continually being reminded of the ill effects of excessive tension and may well be aware of it in ourselves, we are rarely shown just *how* to relax” (Madders, 1973). These sentiments chimed with Lloyd’s reflections on the reasons for starting RforL. As this article has demonstrated, Lloyd and Madders built on and contributed to a longer historical narrative, which responded to but also created that very demand for relaxation training. With relaxation conceptualized as a therapeutic skill that required professional instruction, and with the demand for relaxation training created, Lloyd and Madders, among many others, offered the resources for others to be able to learn the skill.^3^ Lloyd stated in her second newsletter, following RforL acquiring charity status and Madders’ radio series:

In the seven months of our official existence, we have received quite a lot of publicity, bringing in enquiries from all over the country and proving the desperate need for our work. The sad thing is that we can actively help so few people; although we are training teachers … our numbers are as nothing when faced with the demand for our services.

The “desperate need” for relaxation and its development as a means of both counteracting ill-health and promoting wellbeing, grew in tandem. Other organizations such as the Relaxation Society, run by The Revd Geoffrey Harding were in operation at a similar time to when RforL started. As this article has discussed, popular yoga classes routinely included relaxation practice, and a plethora of self-help relaxation books and audio cassettes were in circulation. Relaxation instruction was therefore not as hard to come by as Madders and Lloyd suggested, though there was certainly growing popular demand for courses to learn such techniques within a secular, scientific framework.

Despite the initial concerns over receiving charitable status if relaxation was promoted as both a prophylaxis and remedy, as RforL became further established, prevention remained absolutely essential to the organization’s goals and claims. As Lloyd explained to readers of the NCT Teachers’ Broadsheet in 1979:

Neuro-muscular relaxation enables the individual to lower his arousal and is a vital form of health education. As a preventive therapy it can ease the over-burdened N.H.S. Far too many people already suffer from diseases known to have stress in their origins—hypertension, back pain, coronary disease, ulcers, colitis, migraine, depression, insomnia, phobias, tension headaches, tight muscle pains, nervous indigestion. Many more are heading that way and need to be shown how to unwind.

As well as prevention and therapy for an array of stress-derived illnesses, as the name of the organization signalled, relaxation could and should become embedded in everyday life. As with Jacobson’s differential muscle relaxation techniques and the advice of Boome and Richardson in the 1920s and 1930s, RforL promoted relaxation methods to be used, for example, in the car, at the dentist, at the desk, and in the home. Participants were taught “on-the-spot” techniques that could be adopted until such a time when it was feasible to practise a longer period of restorative “deep relaxation”.

Becoming a relaxed person meant becoming a “balanced” person, which in turn would foster a more harmonious environment through enhancing relationships. Whereas “a tight jaw, shoulders and forehead signal messages of tension and others react accordingly, for anxiety is easily caught”, warned Madders, a relaxed person would have the opposite effect: “When a mother is calm the rest of the household simmers down also”. The same principle held true for businessmen and women, teachers and healthcare professionals. For the health and wellbeing of individuals and society at large, relaxation offered a means of achieving “some inner calm in a hectic changing world” (Madders, 1974). Suffering patients could be given the chance to instead become healthy students of relaxation.

## Conclusion

In 1975, the Harvard cardiologist Herbert Benson published *The Relaxation Response*, which became an international best-seller within weeks. While conducting animal studies on stress and hypertension in the 1960s, which conditioned monkeys to be able to increase and decrease their own blood pressure, he was approached by a group of TM practitioners who claimed their practice enabled them to reduce their blood pressure (Benson, 1975). Benson documented an array of physiological changes through TM, including a drop in heart rate, metabolic rate, brain activity and breathing rate, but considered that these changes were not exclusive to TM. He concluded that a number of practices, including progressive muscle relaxation and prayer from across the world religions, which involved repetition and a passive attitude, could produce the same underlying physiological changes—what he termed the “relaxation response”, the opposite of the “fight or flight” or stress response. Undoubtedly, *The Relaxation Response* significantly increased popular awareness of, and demand for learning relaxation techniques with their widely publicized and scientifically legitimated therapeutic potential. However, as this article has demonstrated, as with RforL, Benson’s success and the public’s receptivity built on a much longer history.

Between 1920 and 1970, significant work had gone into developing therapeutic relaxation as a set of technical skills that had to be taught, learnt and practised and as a framework for everyday living. It had been made relevant to the lives of men and women in distinct ways, and its benefits extendable from the individual to industry, home and state. The major restructuring of the NHS in 1974 did not alter the “health” service’s focus on treating illness and disease. Although relaxation techniques became increasingly practised within clinical settings, such as psychotherapy and physiotherapy, responsibility for maintaining health and enhancing wellbeing lay mainly with individuals. Healthcare consumers primarily had to look outside of the state healthcare system to find and learn techniques such as neuro-muscular relaxation with the hope of preventing and “coping” with a wide range of adverse conditions. In the late 1970s, Thatcherite neoliberal policies accentuated this trend, and relaxation teaching seamlessly entered into the “stress-management” marketplace that burgeoned in the 1980s and beyond.

Into the twenty-first century, health and wellbeing strategies, such as the use of mindfulness-based interventions across clinical and non-clinical settings, reverberate strongly with the calls of earlier therapeutic relaxation proponents and structurally build on their pedagogical frameworks (Nathoo, forthcoming). The appeal of cost-effective, non-pharmaceutical methods of coping with, even thriving within, a “frantic world” (Williams and Penman, 2011) is as relevant now as similar claims from therapeutic relaxation practitioners were almost a century ago.

## Notes

1 See “Amber Lloyd’s early correspondence (1959–1968)”, NCT archive, Wellcome Collection, SA/NCT/B/1/1/1: Box 12; unpublished interviews with Amber Lloyd (10 November 2014) and Prunella Briance (17 January 2012) conducted by Nina Smith, as part of the NCT Oral History Project.

2 For more on the widespread use of tranquillisers in this period see Haggett, 2007; Tone, 2009; Metzel, 2003.

3 As well as Madders, of particular significance for RforL were the widely known teachings of physiotherapist Laura Mitchell, and GP and author Claire Weekes.
